# Community-Based Hypertension Screening to Prevent Stroke in Underserved Urban Neighborhoods in French Guiana: Protocol for a Quasi-Experimental Study

**DOI:** 10.2196/89318

**Published:** 2026-08-27

**Authors:** Devi Rita Rochemont, Mayka Mergeay-Fabre, Aniza Fahrasmane, Claire Boceno, Mathieu Nacher

**Affiliations:** 1 Centre d'Investigation Clinique Antilles Guyane Cayenne, . French Guiana; 2 CHU de Guyane Cayenne French Guiana

**Keywords:** hypertension, stroke, community health workers, early detection of disease, medically underserved area

## Abstract

**Background:**

Hypertension is a leading contributor to stroke and cardiovascular disease in underserved populations.

**Objective:**

This study aims to evaluate a community-based hypertension screening intervention in French Guiana’s precarious urban neighborhoods. This trial will assess whether a structured, community health mediator–led intervention reduces stroke incidence among adults residing in socioeconomically precarious areas of the Communauté d’Agglomération du Centre Littoral (CACL).

**Methods:**

This is a 27-month, monocentric, quasi-experimental study conducted in the CACL, targeting neighborhoods classified as precarious. Adults aged ≥18 years will be included unless they are legally protected or decline participation. Trained mediators will deliver blood pressure screening and health education through mobile units and home visits over a 24-month period. Participants with elevated readings will receive referrals and structured follow-up. The primary outcome is the change in stroke incidence before and after the intervention, using Programme de Médicalisation des Systèmes d’Information (PMSI) hospitalization data. Secondary outcomes include changes in the rates of other cardiovascular and renal events and antihypertensive treatment adherence. Comparisons will use interrupted time series analysis, chi-square tests, and regression models. Missing data will be evaluated, and multiple imputation will be applied as needed.

**Results:**

This study was funded in 2022. Patient enrollment began in September 2023. As of September 4, 2025 (the end of field interventions), 23,289 participants had benefited from the intervention, including 10,901 (46.8%) who were formally enrolled and had completed questionnaires. Follow-up telephone calls were completed until December 2025. Data analysis is expected to be completed in the first quarter of 2026, with primary results anticipated to be published in 2026.

**Conclusions:**

This study will determine whether a scalable, community-mediated hypertension screening intervention reduces stroke incidence in underserved populations.

**Trial Registration:**

ClinicalTrials.gov NCT05814068; https://clinicaltrials.gov/study/NCT05814068

**International Registered Report Identifier (IRRID):**

DERR1-10.2196/89318

## Introduction

### Background

Hypertension, defined as a sustained elevation of systolic blood pressure (BP; ≥130 mm Hg) or diastolic BP (≥80 mm Hg), is a major modifiable risk factor for cardiovascular diseases such as stroke, myocardial infarction, and renal failure [1-6]. It often arises from complex interactions between genetic predisposition and environmental and behavioral factors, and in African Americans with hypertensive nephrosclerosis, the use of angiotensin-converting enzyme inhibitors, such as ramipril, has been shown to slow the rate of glomerular filtration rate (GFR) decline [7]. Globally, hypertension affects more than 1 billion people and is responsible for 13% of all deaths, contributing to 45% of cardiovascular mortality and 51% of stroke-related mortality [3,4,8]. In French Guiana, approximately 350 stroke hospitalizations occur annually, with 68% involving socially vulnerable populations, emphasizing significant health disparities [9]. Community-based strategies for early detection and management have demonstrated effectiveness in diagnosing undetected hypertension and preventing stroke in underserved settings [9-11].

Over the past 2 decades, community-based hypertension screening models have evolved from clinic-centered initiatives to decentralized, multisectoral frameworks aimed at expanding access and mitigating cardiovascular risk [12]. More recently, digital health interventions have emerged as scalable solutions to enhance longitudinal tracking and optimize referral pathways [13]. Concurrently, geospatial mapping and community risk profiling, developed through a virtual, community-based participatory design with a predominantly Black community to address hypertension, now inform the targeted deployment of resources and equitable screening efforts [14]. Collectively, these approaches reflect a shift toward the implementation of evidence-based interventions for hypertension control and stroke prevention, such as salt reduction and stricter tobacco control measures [15].

Despite promising results, several limitations persist in the evaluation of the impact of community-based hypertension screening on stroke incidence [16,17]. Many studies lack robust control groups or use cross-sectional designs, limiting causal inference [18-20]. Self-reported data and office-based BP measurements introduce recall and diagnostic bias, respectively [17,21,22]. Additionally, in socially vulnerable populations, low follow-up rates and limited integration with existing health infrastructure restrict program sustainability [23,24]. Few large-scale studies in low-resource settings, particularly in South America, have analyzed long-term cardiovascular outcomes such as stroke or end-stage renal disease (ESRD), using validated hospitalization data [25,26]. Moreover, evidence is sparse on adherence to referrals and longitudinal prescription patterns following screening, indicating an underexplored area for assessing the intervention’s impact [27].

To address the critical gap in longitudinal data on stroke prevention in low-resource settings, this prospective, community-based, quasi-experimental study was designed to evaluate the impact of decentralized hypertension screening on stroke incidence among adults aged ≥18 years residing in socioeconomically precarious neighborhoods within the Communauté d’Agglomération du Centre Littoral (CACL) region of French Guiana. Using mobile health stations and home visits, trained health mediators will conduct standardized BP assessments and educational interventions, linking individuals with elevated readings to appropriate health care services. The main outcome—hospital-based stroke incidence—will be measured using national administrative datasets, offering novel insights into population-level vascular risk mitigation in underserved communities.

### Objectives

#### Primary Objective

The primary objective is to determine whether a community-based hypertension screening and referral intervention delivered by trained health mediators is superior to standard health service delivery in reducing the incidence of stroke (*accidents vasculaires cérébraux* [AVC]) over a 2-year period, compared to the 5-year preintervention period, among adults residing in the CACL region of French Guiana.

#### Secondary Objectives

The secondary objectives are to evaluate the effectiveness of the intervention in reducing the incidence and mortality of other major cardiovascular and renal outcomes, including acute coronary syndrome (ACS), transient ischemic attack (TIA), peripheral artery disease (PAD), aneurysm, and ESRD; to assess participants’ adherence to referrals and satisfaction with the program; and to evaluate changes in screening coverage and antihypertensive treatment patterns. Comparisons will also be conducted between residents of targeted precarious neighborhoods and those living in nonintervention zones within and outside the intervention area.

## Methods

### Study Design and Setting

**Figure 1:**
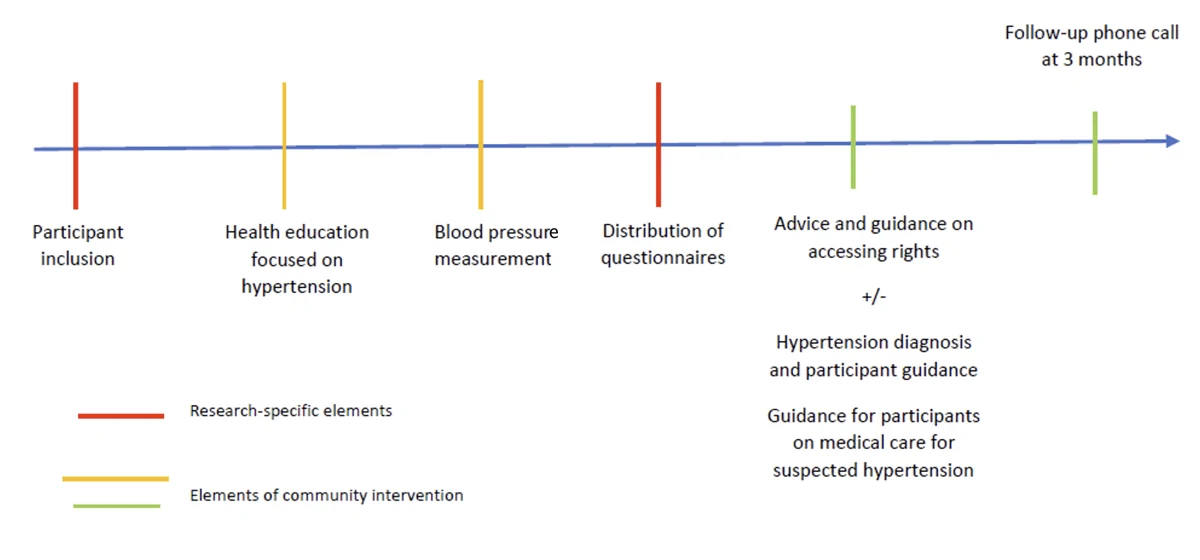
Participant workflow during the study.

**Figure 2:**
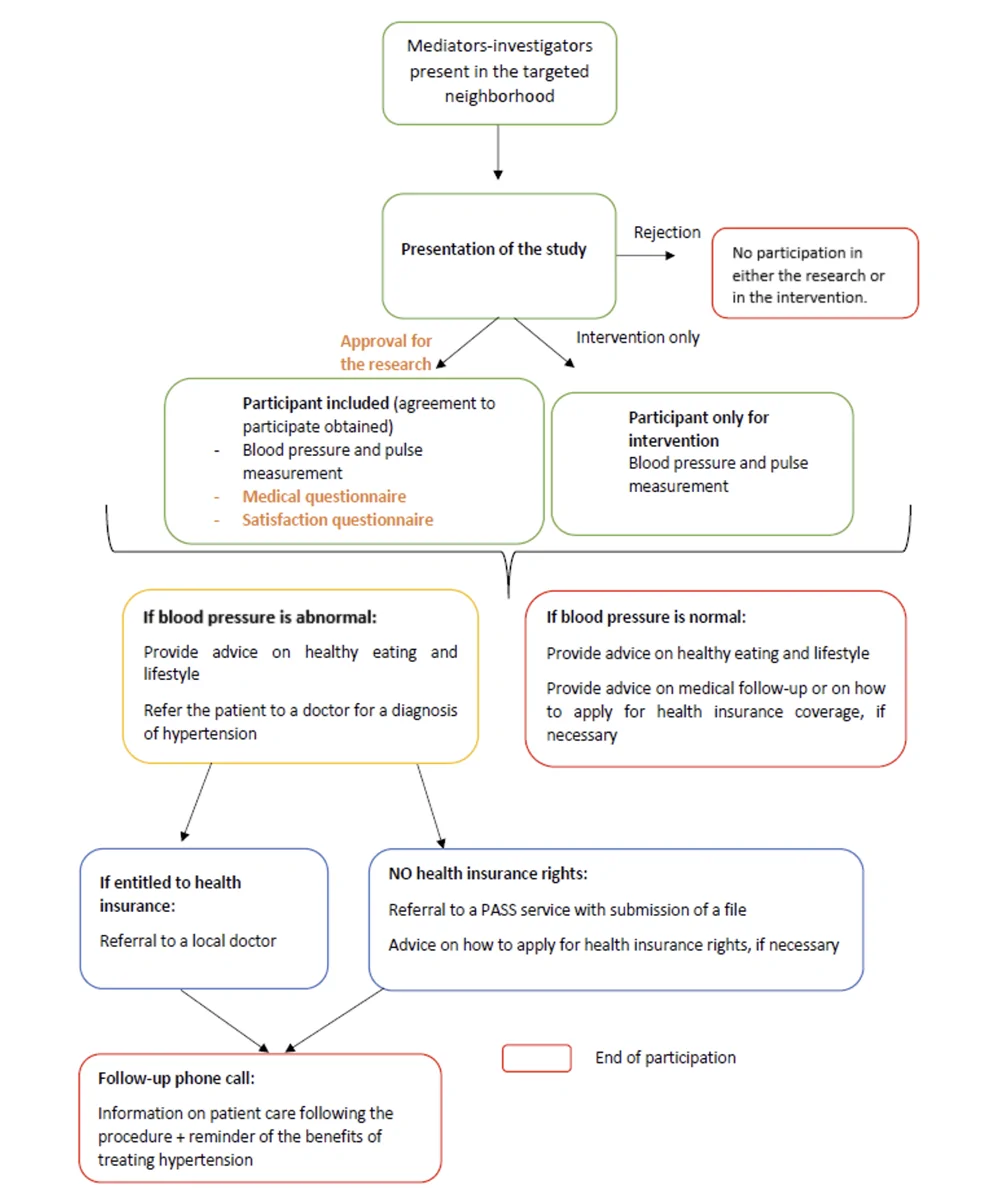
Study flow diagram. PASS: Permanence d’Accès aux Soins de Santé.

This study is a monocentric, prospective, quasi-experimental intervention conducted within the CACL of French Guiana, comprising the communes of Cayenne, Matoury, Rémire-Montjoly, Roura, Montsinéry-Tonnegrande, and Macouria. The intervention targets socioeconomically precarious neighborhoods and aims to evaluate the effectiveness of a community-based hypertension screening initiative delivered through trained health mediators. The study spans a total of 27 months, comprising a 24-month intervention period followed by a 3-month monitoring and follow-up phase (Figures 1 and 2). The intervention is designed to be inclusive and is accessible to all residents within the targeted neighborhoods, regardless of their eligibility for the study or their decision to participate in the research.

All interested participants receive a brief health education session covering hypertension awareness, associated dietary and lifestyle recommendations, and guidance on accessing health care entitlements. Participants may also be referred to other health care facilities or professionals in the event of health concerns other than hypertension. Subsequently, each participant undergoes standardized BP measurement using 3 readings spaced 1 to 2 minutes apart, including at least 1 reading from each arm, in accordance with European guidelines. If BP values suggest possible hypertension, participants are referred for medical evaluation either to a local physician or to the Permanence d’Accès aux Soins de Santé (PASS) based on their insurance status.

Suspected cases are provided with a formal referral document detailing the observed metrics to facilitate continuity of care within the health care system.

Participants identified with elevated BP are contacted by telephone at 3 months after inclusion (with an allowable variation of ±1 week) to assess diagnostic follow-through, treatment initiation, and compliance with medical recommendations. During this follow-up, mediators reinforce the importance of ongoing care if lapses are identified.

In parallel, after the mediators have provided oral information about the study, they verify the inclusion (age ≥18 years, residence in the CACL, and absence of legal protection) and exclusion criteria and obtain the participant’s nonopposition to participation. For these participants, an additional structured questionnaire is administered to collect sociodemographic and anthropometric data, cardiovascular history, prior diagnoses, medication adherence, barriers to health care access, and a satisfaction survey.

Withdrawal from the study is permitted at any time without justification and does not affect access to usual medical care. Investigators may also discontinue participation if continued participation is deemed not to be in the participant’s best interest. Reasons for withdrawal or loss to follow-up will be recorded when available.

Figure 2 outlines the stepwise procedure for participant inclusion, assessment, and subsequent actions based on BP results and health insurance status, including interventions, referrals, and follow-up steps.

### Participants

Eligible participants will be adults aged ≥18 years residing within the CACL territory, particularly in neighborhoods identified as precarious based on urban, social, or environmental exclusion. There are no restrictions regarding sex or nationality. Inclusion will be based on residency and age only (Textbox 1). Individuals who explicitly express opposition to participation or who are under legal protection (eg, guardianship or curatorship) will be excluded from data collection. Recruitment will occur within ambulatory settings across targeted neighborhoods, where health mediators will actively engage the population through fixed locations, door-to-door outreach, and dedicated information campaigns involving flyers, posters, and local radio messages. Translation support and multilingual team members will ensure accessibility for participants regardless of their native language.

**Inclusion and exclusion criteria.**

**Inclusion criteria**

- Adults aged ≥18 years
- Individuals residing in the Communauté d’Agglomération du Centre Littoral

**Exclusion criteria**

- Individuals who explicitly refuse to participate in the study
- Individuals under legal protection such as guardianship or conservatorship

### Randomization and Blinding

Because this study is nonrandomized and uses a quasi-experimental design, no randomization procedures will be used. Participants will be naturally exposed to the intervention as a function of their geographic residence. To limit assessment bias, the primary outcomes will be based on objective administrative data from hospital discharge records and national health insurance datasets. These datasets are not influenced by participants’ awareness of their inclusion in the study.

### Intervention or Treatment

The intervention will consist of a comprehensive, community-delivered hypertension screening and education program. It will be implemented by 12 trained health mediators organized into 3 teams of 3 to 4 mediators each. The protocol stipulates that each mediator can screen approximately 10 people per day, representing a target of 2000 adults per month. These mediators, certified through a university-level diploma in health mediation or with equivalent field experience, will engage directly with residents in all precarious neighborhoods within the CACL. Each neighborhood will receive at least 1 intervention visit during the 24-month intervention period. The number of visits per neighborhood will be determined based on population size and accessibility. The goal is comprehensive coverage of an estimated population of 50,000 adults in the target neighborhoods.

To maximize reach, each visit will last 1 to 2 days, with mediators establishing fixed screening posts and conducting door-to-door outreach. The quantification of coverage will not be based solely on the number of individuals screened but on the number of neighborhoods fully investigated by the mediators. The intervention will be considered complete for a neighborhood when the minimum number of planned visits has been conducted, when mediators have systematically covered all streets or sectors through door-to-door approaches, or when repeated visits yield diminishing returns.

Given population mobility and the informal nature of precarious settlements, we acknowledge that precise denominator estimation is challenging. Mediators will set up mobile stations or conduct home visits to measure BP triadically according to European guidelines, provide personalized health education focused on hypertension and lifestyle modification, assess health insurance coverage, and guide participants with elevated BP to appropriate health care professionals or social services (eg, general practitioners or the PASS unit). Follow-up will include a structured telephone call 3 months later to assess completion of the medical referral process.

### Mediator Training and Certification

All 12 health mediators completed either a university diploma in health mediation (*diplôme universitaire de médiation en santé*, University of French Guiana) or possess equivalent certified field experience. Prior to intervention deployment, mediators received specific training consisting of clinical training on BP measurement techniques and device use; training on hypertension pathophysiology, complications, and dietary and lifestyle recommendations adapted to local cultural contexts; training on prevention posture, mediation, and individual counseling techniques; and supervised practice sessions with feedback. Mediators work in teams of 3 to ensure multilingual coverage (French, Creole, Portuguese, Spanish, Arabic, English, and indigenous languages as needed). Each team is equipped with validated BP monitors, educational materials, referral forms, and mobile phones for coordination and follow-up.

### Outcome Measures

The primary outcome will be the change in the incidence of stroke comparing the 5-year preintervention period with the 2-year period following the start of the intervention, as recorded in the Programme de Médicalisation des Systèmes d’Information (PMSI) database. Secondary outcomes will include changes in incidence of ACS [28], TIA [29], PAD [30], aneurysm, and ESRD [31] (*insuffisance rénale terminale* [IRT]), as well as all-cause cardiovascular mortality within 1, 2, and 3 years after the intervention. ACS refers to a spectrum of urgent cardiac conditions caused by a sudden reduction in blood flow to the heart muscle due to a partial or complete blockage of the coronary arteries. This group of conditions includes unstable angina and non–ST-elevation myocardial infarction (NSTEMI), both of which require prompt diagnosis and individualized treatment strategies to prevent further cardiac damage and improve patient outcomes.

ESRD represents the final and most advanced stage of chronic kidney disease, defined by a severely reduced GFR of less than 15 mL/min/1.73 m^2^. At this stage, patients require kidney replacement therapy, which includes either long-term dialysis or kidney transplantation. ESRD is a chronic condition that typically arises in the context of long-standing comorbidities, notably hypertension.

PAD is a circulatory condition characterized by the narrowing or blockage of peripheral arteries, most commonly in the lower limbs, due to the buildup of atherosclerotic plaques. This reduced blood flow can lead to symptoms such as leg pain during walking (claudication), numbness, or even tissue damage in severe cases. Importantly, PAD is not confined to the extremities; it reflects a systemic atherosclerotic process that affects multiple vascular beds. As such, PAD serves as a clinical marker for widespread vascular dysfunction and is strongly associated with an elevated risk of major cardiovascular events, including myocardial infarction and stroke.

Additional secondary end points will assess participants’ adherence to medical referrals, satisfaction with the program, the number of BP screenings completed, the number of new or rediagnosed hypertensive cases, and changes in antihypertensive prescription patterns using health insurance data. Outcome assessments will be based solely on administrative records and structured questionnaires, minimizing subjectivity and measurement variability.

### Data Collection Tools

BP will be measured using validated electronic arm-cuff devices following standardized procedures: 3 measurements at 1- to 2-minute intervals after 5 minutes of rest, recording systolic and diastolic pressure in both arms following the European Society of Hypertension guidelines. Mediators document results immediately on structured forms and provide participants with referral slips if indicated. Sociodemographic, anthropometric, and medical history data will be collected using a paper-based questionnaire estimated to require 30 minutes per participant. The questionnaire included demographic data (age, sex, country of birth, languages spoken, and duration of residence), medical history (previous hypertension diagnosis, current medications, and cardiovascular events), health care access (insurance status, usual source of care, and barriers to access), and lifestyle factors (smoking, alcohol consumption, physical activity, and dietary habits). For participants with suspected hypertension, a standardized 3-month phone follow-up will be performed using a semistructured script to capture referral completion and treatment adherence. Administrative data on hospitalizations, diagnoses, and prescriptions will be accessed from the regional PMSI [32] and national health insurance databases. PMSI is a French hospital discharge database that compiles standardized clinical and administrative information on all inpatient stays in both public and private hospitals. It includes data such as patient demographics, diagnoses, medical procedures, and length of hospital stay. This system enables the construction of patient cohorts based on variables such as age, sex, comorbidities, and disease severity. Data are routinely collected from health care facilities, ensuring comprehensive population coverage and minimizing selection bias. Its use is governed by strict ethical and data protection regulations. No biological samples will be collected.

### Data Quality and Monitoring Procedures

Data collection forms are reviewed daily by team supervisors for completeness and consistency. Electronic data entry into the Ennov Clinical database is performed by trained research assistants with automatic range and consistency checks. There is regular verification of different aspects, including the appropriate completion of nonopposition documentation; the accuracy of transcription from paper forms to the electronic database; proper storage of source documents; adherence to protocol procedures; and finally, verification of a random sample of 10% of participant records against database entries.

### Safety Assessment

The intervention carries minimal risk. All procedures, including BP measurement and health interviews, are noninvasive and in line with routine prevention practices. No pharmacological treatments, invasive diagnostics, or biological sample collection are involved. Adverse events are not expected; however, participants experiencing persistently dangerously elevated BP (eg, >180/110 mm Hg) will be referred immediately for urgent care. No data safety monitoring board (DSMB) has been implemented because of the low-risk classification (category 3) of the intervention.

### Statistical Analysis

Statistical analyses will be conducted using Stata (version 19; StataCorp). Continuous variables will be described using means and SDs when normally distributed; for skewed distributions, medians and IQR will be reported. Normality will be assessed using visual inspection of histograms and quantile-quantile plots and confirmed with the Shapiro-Wilk test.

Comparisons of continuous variables between groups (eg, before vs after the intervention and intervention vs control regions) will be performed using the Student *t* test or the Mann-Whitney *U* test, as appropriate. The Student *t* test is a statistical method used to determine whether the means of 2 independent groups are significantly different from each other. It assumes that the data in each group are approximately normally distributed and that the variances are equal or similar.

Categorical variables will be summarized as absolute frequencies and percentages. Between-group comparisons will be conducted using chi-square tests or Fisher exact tests.

All statistical comparisons will be 2-sided, and results will be considered statistically significant if *P*<.05. Corresponding 95% CIs will be reported for all estimated parameters.

**Figure 3:**
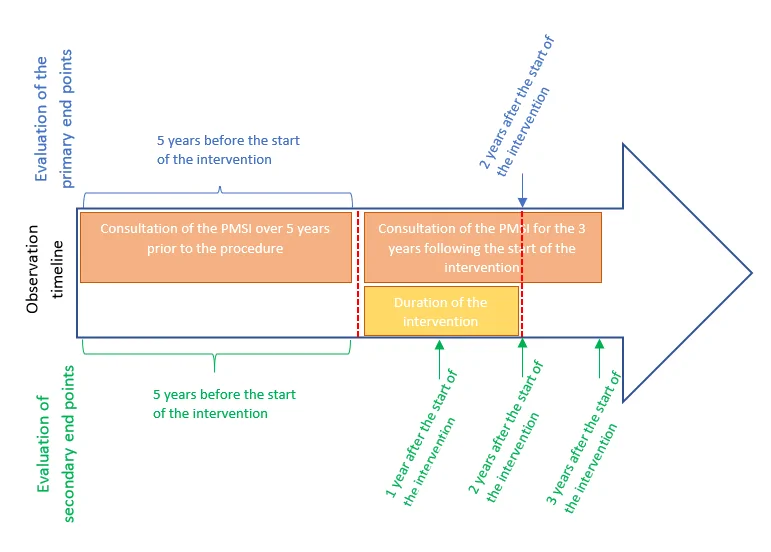
Study outcome assessment timeline. PMSI: Programme de Médicalisation des Systèmes d’Information.

The primary outcome analysis will assess the difference in stroke incidence before and after the 2-year intervention period among residents of precarious neighborhoods within the CACL of French Guiana. Stroke incidence, derived from PMSI hospitalization data, will be compared across the 2 periods using the chi-square test. To evaluate temporal trends and detect structural breaks attributable to the intervention, segmented regression of interrupted time series (ITS) [33] data will be applied. The ITS model will estimate the immediate change in level and the change in slope after the intervention, while adjusting for autocorrelation and seasonality as necessary based on model diagnostics (Figure 3).

ITS analysis is a quasi-experimental design used to evaluate the longitudinal effect of an intervention by analyzing data collected at multiple time points before and after the intervention is implemented. This method enables the detection of both abrupt changes in outcome level and gradual changes in trend that may result from the intervention. ITS is particularly valuable when randomized controlled trials are not feasible, because it allows causal inference from observational data by controlling for underlying trends and temporal confounders. By modeling the preintervention trajectory and comparing it to the postintervention pattern, ITS helps determine whether observed changes are likely attributable to the intervention rather than to external factors or natural fluctuations over time.

Secondary outcomes—including the incidence of ACS, TIA, peripheral arterial disease, aneurysms, and ESRD, as well as cardiovascular and renal mortality—will also be analyzed using ITS models. ITS will help determine whether these outcomes exhibit a significant change in their rate or trajectory after the intervention. Subanalyses will compare trends in populations residing in intervention and nonintervention neighborhoods within the CACL and between the intervention region and other areas of French Guiana where the intervention was not deployed. Comparisons will further be stratified by socioeconomic status markers (eg, French universal health coverage, *aide médicale de l'état* [AME] eligibility, or absence of coverage) when available in the source databases.

For participant-level data obtained through questionnaires (eg, sociodemographic characteristics, adherence indicators, and antihypertensive treatment uptake), analyses will include paired comparisons for repeated measures, using the McNemar test [34] or the Wilcoxon signed-rank tests [35] depending on the data type and distribution.

McNemar test is a nonparametric method used to analyze paired nominal data, particularly in situations where the same participants are measured at 2 time points, such as before and after an intervention. It is designed to detect changes in proportions by focusing exclusively on discordant pairs—cases where the response changed between the 2 time points—while ignoring concordant pairs in which no change occurred. This makes it especially suitable for evaluating shifts in binary outcomes within matched or repeated-measures designs, where observations are not independent.

Trends in antihypertensive prescription volumes, obtained from health insurance reimbursement data, will also be evaluated over the preintervention and postintervention periods using ITS models to assess the effect of the intervention at the population level.

Missing data will be systematically evaluated for all variables. For descriptive analyses, the number of participants with available data will be reported. For analytical purposes, the mechanism of missingness will be assessed (eg, missing completely at random, missing at random, or missing not at random). If data are found to be missing at random, multiple imputation via chained equations (MICE) [36] will be considered for inclusion in multivariable and ITS analyses. MICE is a flexible and iterative method for handling missing data by creating multiple complete datasets. It works by modeling each variable with missing values conditionally on all other variables in the dataset, using appropriate regression models depending on the type of variable (eg, linear for continuous variables and logistic for binary variables). This chained approach allows the preservation of complex relationships among variables and accommodates different data types, improving the validity and efficiency of statistical inferences drawn from incomplete data.

As a sensitivity check, complete-case analyses will also be performed. The influence of missing data on primary and secondary outcomes will be explicitly reported.

Sample size calculations were conducted using the following two complementary approaches.

The primary approach uses the chi-square test; approximately 350 patients with stroke are admitted annually to the Centre Hospitalier de Cayenne, with 68% (n≈238) occurring among socioeconomically precarious patients. Assuming the intervention leads to a 30% reduction in stroke incidence within this population—consistent with previous international interventions—a postintervention count of approximately 167 strokes per year (a reduction from 238) is anticipated. To detect this effect with 90% power at a 5% significance level (α=.05) using a 2-sided chi-square test comparing proportions before and after the intervention, a minimum of 367 cumulative postintervention stroke cases over a 2-year period is required.

Second, a segmented regression (ITS) analysis will be used to complement the chi-square test by examining both immediate level changes and slope changes after the intervention. On the basis of 60 monthly preintervention observations (5 years) and 24 monthly postintervention observations (2 years), and assuming autocorrelation (ρ=0.3-0.5) typical of health administrative data, this design provides adequate power (>80%) to detect a change in slope corresponding to a 20% to 30% reduction in incidence, assuming a baseline coefficient of variation of 15% to 20% in monthly stroke counts.

The chi-square test provides a straightforward test of the primary hypothesis, while ITS analysis accounts for underlying temporal trends and potential confounders, offering a more nuanced understanding of intervention effects. Both analyses will be reported, with ITS serving as the primary confirmatory analysis and the chi-square test as a sensitivity check.

### Ethical Considerations

The study protocol has been reviewed and approved by the Comité de Protection des Personnes (CPP) South Mediterranean I, on January 11, 2023 (reference number SI 22.01568.000191 and national number ID-RCB 2022-A026000-43; category 3 minimal risk research). The study was registered on ClinicalTrials.gov on April 24, 2023 (NCT05814068).

All participants receive oral information about the study’s objectives, procedures, the voluntary nature of participation, and their rights. Written informed consent will not be sought because of high illiteracy rates in the target population. Rather, trained mediators document participants’ nonopposition to participation after ensuring comprehension through an oral explanation in the participant’s preferred language. Multilingual mediators and translation services ensure accessibility. The study adheres to the ethical principles outlined in the Declaration of Helsinki and complies with French data protection legislation (Loi Informatique et Libertés, modified on August 6, 2004) and the European General Data Protection Regulation (GDPR, 2016/679). A Data Protection Impact Assessment was completed prior to study initiation. Data are deidentified before analysis using a unique study identifier, with the correspondence table stored securely and separately from the research data. Access is restricted to authorized personnel only. Participants may withdraw at any time without justification and without affecting access to usual medical care. The promoter (Centre Hospitalier de Cayenne) has declared this research to the Commission Nationale de l’Informatique et des Libertés (CNIL) under simplified declaration procedures for health research (*méthodologie de référence* MR-003), with the engagement dated December 21, 2021.

## Results

This study was funded in 2022 and registered on ClinicalTrials.gov in April 2023 (NCT05814068). Recruitment for this study began in September 2023 and concluded on September 4, 2025, marking the end of the 24-month intervention period. During this period, 23,289 participants benefited from the intervention (ie, received at least 1 BP measurement and health education session), including 10,901 (46.8%) who completed the full enrollment questionnaire. Three-month telephone follow-up for participants with elevated BP was conducted as scheduled and completed by December 2025. Case report form (CRF) data entry and quality monitoring procedures were in progress as of February 2026, with database lock planned for March 2026 and primary outcome analysis expected between April 2026 and May 2026.

## Discussion

### Principal Anticipated Findings

This community-based hypertension screening intervention in French Guiana’s precarious neighborhoods is anticipated to demonstrate a significant reduction in stroke incidence among socioeconomically vulnerable populations. On the basis of the comprehensive deployment of trained health mediators who screened >23,000 participants across 32 neighborhoods, we expect to observe a 20% to 30% reduction in stroke incidence in the 2-year postintervention period compared to the 5-year baseline period. If confirmed, this would represent one of the largest quasi-experimental demonstrations of stroke prevention through community-based hypertension screening in a low- to middle-income setting in Amazonia. It is anticipated that the intervention will also result in a significant decline in other cardiovascular and renal events compared to the preintervention period.

### Comparison to Prior Work

By targeting underserved neighborhoods and using trained health mediators, the project introduces an innovative strategy that complements current health service models.

The anticipated outcomes of this community-based hypertension screening intervention are expected to align with previous studies indicating that population-level hypertension control can reduce stroke incidence significantly [37]. It is anticipated that the proposed intervention’s impact will meet or exceed this reduction given its specific focus on vulnerable populations with a high baseline prevalence of uncontrolled hypertension and limited access to care [38-40].

Unlike prior interventions that often lacked integration into existing community health infrastructure, this project’s use of local health mediators may enhance referral adherence and treatment initiation, addressing a common limitation cited in prior studies, in which more than 40% of referred patients failed to access follow-up care [24,27]. Furthermore, although previous programs reported limited sustainability because of their reliance on nonintegrated, short-term staff, this study’s design aims to improve scalability through embedded community-based actors [24]. Thus, the expected findings will provide valuable comparative data for existing models and address gaps in stroke prevention strategies, particularly in socially underserved urban settings in low- and middle-income regions [37].

Beyond disease incidence, the study will assess adherence to medical referrals, satisfaction with the program, and changes in antihypertensive treatment patterns, with the objective of generating scalable insights for health policy and population-level prevention strategies.

### Strengths and Limitations

This study has several important strengths. First, the use of comprehensive administrative hospitalization data (PMSI) for the primary outcome eliminates selection bias in outcome ascertainment, as all stroke hospitalizations in the region are systematically captured regardless of participants’ awareness of the intervention. Second, the large-scale, community-based approach targets an entire socioeconomically vulnerable population across neighborhoods over 24 months, providing real-world evidence of intervention feasibility and reach. Third, the use of trained community health mediators from the same cultural backgrounds as the target populations enhances intervention acceptability and reduces barriers related to language and trust. Fourth, the 5-year preintervention baseline period and ITS analysis allow a robust assessment of temporal trends and intervention effects while controlling for underlying changes in stroke incidence.

However, several limitations must be acknowledged. The quasi-experimental design without randomization means that temporal confounders (such as concurrent public health campaigns, changes in health care access, or secular trends in treatment patterns) may influence outcomes independently of the intervention. Although we will attempt to control for these through comparison with nonintervention regions in French Guiana, unmeasured confounding remains possible. Reliance on administrative datasets, while ensuring comprehensive capture, may be subject to coding inaccuracies or changes in diagnostic practices over time. However, the use of validated *International Statistical Classification of Diseases and Related Health Problems, 10th revision* (ICD-10) codes for stroke diagnosis and the consistency of PMSI reporting requirements over the study period minimize this concern. Implementation heterogeneity across neighborhoods (varying in size, accessibility, and population composition) may introduce variability in intervention dose and reach. We will document coverage rates by neighborhood and explore heterogeneity in subgroup analyses, but differences in how intensively the intervention was implemented across neighborhoods may influence the results. For secondary outcomes assessed through participant questionnaires and telephone follow-up (eg, referral adherence, treatment initiation, and satisfaction), self-reported data may be subject to social desirability and recall biases. Additionally, loss to follow-up during the 3-month telephone interviews may affect internal validity for these secondary end points, although we plan to use multiple imputation techniques and will report sensitivity analyses restricted to complete cases. These limitations, however, do not affect the primary outcome analysis, which relies exclusively on objective administrative data. Finally, the exclusion of individuals under legal protection (eg, guardianship) is required by French research regulations but may reduce generalizability to populations with severe cognitive impairment or psychiatric conditions, groups that may also experience an elevated risk of cardiovascular disease. Despite these limitations, this study addresses a critical gap in stroke prevention research in underserved populations and will provide valuable evidence on the effectiveness and scalability of community-based hypertension screening in resource-limited settings.

### Future Directions and Dissemination Plan

Results will be disseminated through multiple channels, including peer-reviewed publications in open-access journals; presentations at regional (French territories) and international conferences on cardiovascular prevention and health equity; policy briefs for French Guiana health authorities and the Agence Régionale de Santé; and community feedback sessions in participating neighborhoods. A dedicated stakeholder workshop will be organized in Cayenne in late 2026 to discuss scalability and sustainability with local health authorities, nongovernmental organizations (NGOs), and community representatives.

## Appendix

Multimedia Appendix 1 Peer-review report 1 from Programme de recherche sur la performance du système de soins (PREPS), Ministry of Health (France).

Multimedia Appendix 2 Peer-review report 2 from Programme de recherche sur la performance du système de soins (PREPS), Ministry of Health (France).

Multimedia Appendix 3 Peer-review report 3 from Programme de recherche sur la performance du système de soins (PREPS), Ministry of Health (France).

## Abbreviations

ACS: acute coronary syndrome
AME: aide médicale de l'état
AVC: accidents vasculaires cérébraux
BP: blood pressure
CACL: Communauté d’Agglomération du Centre Littoral
CNIL: Commission Nationale de l’Informatique et des Libertés
CPP: Comité de Protection des Personnes
CRF: case report form
DSMB: data safety monitoring board
ESRD: end-stage renal disease
GDPR: General Data Protection Regulation
GFR: glomerular filtration rate
ICD-10: International Statistical Classification of Diseases and Related Health Problems, 10th revision
IRT: insuffisance rénale terminale
ITS: interrupted time series
MICE: multiple imputation via chained equations
NGO: nongovernmental organization
NSTEMI: non–ST-elevation myocardial infarction
PAD: peripheral artery disease
PASS: Permanence d’Accès aux Soins de Santé
PMSI: Programme de Médicalisation des Systèmes d’Information
TIA: transient ischemic attack

## References

[1] Unger T, Borghi C, Charchar F, Khan NA, Poulter NR, Prabhakaran D, Ramirez A, Schlaich M, Stergiou GS, Tomaszewski M, Wainford RD, Williams B, Schutte AE (2020). 2020 International Society of Hypertension global hypertension practice guidelines. Hypertension.

[2] Kitagawa K (2022). Blood pressure management for secondary stroke prevention. Hypertens Res.

[3] Xia T, Zhao F, Nianogo RA (2022). Interventions in hypertension: systematic review and meta-analysis of natural and quasi-experiments. Clin Hypertens.

[4] Chowdhury MZ, Rahman M, Akter T, Akhter T, Ahmed A, Shovon MA, Farhana Z, Chowdhury N, Turin TC (2020). Hypertension prevalence and its trend in Bangladesh: evidence from a systematic review and meta-analysis. Clin Hypertens.

[5] Mills KT, Stefanescu A, He J (2020). The global epidemiology of hypertension. Nat Rev Nephrol.

[6] Carey RM, Moran AE, Whelton PK (2022). Treatment of hypertension: a review. JAMA.

[7] Sica DA (2003). The African American Study of Kidney Disease and Hypertension (AASK) trial: what more have we learned?. J Clin Hypertens (Greenwich).

[8] Schmidt BM, Durao S, Toews I, Bavuma CM, Hohlfeld A, Nury E, Meerpohl JJ, Kredo T (2020). Screening strategies for hypertension. Cochrane Database Syst Rev.

[9] Hart JT (2001). Commentary: can health outputs of routine practice approach those of clinical trials?. Int J Epidemiol.

[10] Bosu WK, Bosu DK (2021). Prevalence, awareness and control of hypertension in Ghana: a systematic review and meta-analysis. PLoS One.

[11] Oliveros E, Patel H, Kyung S, Fugar S, Goldberg A, Madan N, Williams KA (2020). Hypertension in older adults: assessment, management, and challenges. Clin Cardiol.

[12] Deo S, Singh P (2021). Community health worker-led, technology-enabled private sector intervention for diabetes and hypertension management among urban poor: a retrospective cohort study from large Indian metropolitan city. BMJ Open.

[13] Liu F, Song T, Yu P, Deng N, Guan Y, Yang Y, Ma Y (2023). Efficacy of an mHealth app to support patients' self-management of hypertension: randomized controlled trial. J Med Internet Res.

[14] Robles MC, Newman MW, Doshi A, Bailey S, Huang L, Choi SJ, Kurien C, Merid B, Cowdery J, Golbus JR, Huang C, Dorsch MP, Nallamothu B, Skolarus LE (2022). A physical activity just-in-time adaptive intervention designed in partnership with a predominantly Black community: virtual, community-based participatory design approach. JMIR Form Res.

[15] Rahim HF, Sibai A, Khader Y, Hwalla N, Fadhil I, Alsiyabi H, Mataria A, Mendis S, Mokdad AH, Husseini A (2014). Non-communicable diseases in the Arab world. Lancet.

[16] Tran HH, Thu A, Twayana AR, Fuertes A, Gonzalez M, Mehta KA, Basta M, James M, Elias D, Figaro YM, Islek D, Lo A, Frishman WH, Aronow WS (2025). Improving access and outcomes in cardiovascular care for racial and ethnic minorities. Cardiol Rev.

[17] Hao G, Chen Z, Wang X, Zhang L, Kang Y, Zheng C, Chen L, Wang Z, Gao R (2022). Evaluation of the community-based hypertension management programs in China. Front Public Health.

[18] Ioannidis JP (2005). Why most published research findings are false. PLoS Med.

[19] Douine M, Lambert Y, Plessis L, Bardon T, Adenis A, Nacher M, Suarez-Mutis M, Vreden S, Mathieu O, Sanna A (2025). A cross-sectional study assessing mercury exposure and poisoning among workers in informal gold mines in French Guiana. BMJ Open.

[20] McGuire H, Van TB, Thi Thu HL, Nguyen Thanh H, Murray M, Shellaby J, Aerts A, George R, Hodges M (2022). Improving hypertension awareness and management in Vietnam through a community-based model. Sci Rep.

[21] Myers MG (2018). Automated office blood pressure measurement. Korean Circ J.

[22] Matsumoto K, Jin Z, Homma S, Elkind MS, Schwartz JE, Rundek T, Mannina C, Ito K, Sacco RL, Di Tullio MR (2021). Office, central, and ambulatory blood pressure for predicting first stroke in older adults: a community-based cohort study. Hypertension.

[23] McManus RJ, Mant J, Roalfe A, Oakes RA, Bryan S, Pattison HM, Hobbs FD (2005). Targets and self monitoring in hypertension: randomised controlled trial and cost effectiveness analysis. BMJ.

[24] Kobashi Y, Haque SE, Sakisaka K, Amir I, Kaneko M, Mutahara M, Mubassara S, Kashem A, Tsubokura M (2024). Community-based intervention for managing hypertension and diabetes in rural Bangladesh. Trop Med Health.

[25] Ordunez P, Mize V, Barbosa M, Legetic B, Hennis AJ (2015). A rapid assessment study on the implementation of a core set of interventions to improve cardiovascular health in Latin America and the Caribbean. Glob Heart.

[26] Clarke L, Castor-Newton MJ, Jalles C, Lapeyre-Mestre M, Gardette V (2024). Potentially avoidable hospitalizations and associated factors among older people in French Guiana using the French National Health Data System. Int J Qual Health Care.

[27] Madela S, James S, Sewpaul R, Madela S, Reddy P (2020). Early detection, care and control of hypertension and diabetes in South Africa: a community-based approach. Afr J Prim Health Care Fam Med.

[28] Howard BG, Wenger NK (2016). Review of the 2014 AHA/ACC guideline for the management of patients with non-ST-elevation acute coronary syndromes: what is new and why?. Curr Cardiovasc Risk Rep.

[29] Easton JD, Saver JL, Albers GW, Alberts MJ, Chaturvedi S, Feldmann E, Hatsukami TS, Higashida RT, Johnston SC, Kidwell CS, Lutsep HL, Miller E, Sacco RL (2009). Definition and evaluation of transient ischemic attack: a scientific statement for healthcare professionals from the American Heart Association/American Stroke Association Stroke Council; Council on Cardiovascular Surgery and Anesthesia; Council on Cardiovascular Radiology and Intervention; Council on Cardiovascular Nursing; and the Interdisciplinary Council on Peripheral Vascular Disease. The American Academy of Neurology affirms the value of this statement as an educational tool for neurologists. Stroke.

[30] Itoga NK, Tawfik DS, Lee CK, Maruyama S, Leeper NJ, Chang TI (2018). Association of blood pressure measurements with peripheral artery disease events. Circulation.

[31] Verberne WR, Das-Gupta Z, Allegretti AS, Bart HA, van Biesen W, García-García G, Gibbons E, Parra E, Hemmelder MH, Jager KJ, Ketteler M, Roberts C, Al Rohani M, Salt MJ, Stopper A, Terkivatan T, Tuttle KR, Yang CW, Wheeler DC, Bos WJ (2019). Development of an international standard set of value-based outcome measures for patients with chronic kidney disease: a report of the International Consortium for Health Outcomes Measurement (ICHOM) CKD Working Group. Am J Kidney Dis.

[32] Chevreul K, Berg Brigham K, Durand-Zaleski I, Hernandez-Quevedo C (2015). France: health system review. Health Syst Transit.

[33] Xu J, Yin X, Jiang T, Wang S, Wang D (2023). Effects of air pollution control policies on intracerebral hemorrhage mortality among residents in Tianjin, China. BMC Public Health.

[34] McNemar Q (1947). Note on the sampling error of the difference between correlated proportions or percentages. Psychometrika.

[35] Wilcoxon F (1945). Individual comparisons by ranking methods. Biometr Bull.

[36] Rubin DB (1989). Multiple Imputation for Nonresponse in Surveys.

[37] Nowrin I, Mehareen J, Bhattacharyya DS, Saif-Ur-Rahman KM (2023). Community-based interventions to prevent stroke in low and middle-income countries: a systematic review. Health Sci Rev.

[38] Van Melle A, Cropet C, Parriault MC, Adriouch L, Lamaison H, Sasson F, Duplan H, Richard JB, Nacher M (2019). Renouncing care in French Guiana: the national health barometer survey. BMC Health Serv Res.

[39] Valmy L, Gontier B, Parriault MC, Van Melle A, Pavlovsky T, Basurko C, Grenier C, Douine M, Adenis A, Nacher M (2016). Prevalence and predictive factors for renouncing medical care in poor populations of Cayenne, French Guiana. BMC Health Serv Res.

[40] Rochemont DR, Lemenager P, Franck Y, Farhasmane A, Sabbah N, Nacher M (2021). The epidemiology of acute coronary syndromes in French Guiana. Ann Cardiol Angeiol (Paris).

