# Supplementary material for: Community-Based Hypertension Screening to Prevent Stroke in Underserved Urban Neighborhoods in French Guiana: Protocol for a Quasi-Experimental Study
Source: JMIR Res Protoc. 2026 Aug 27;15:e89318. doi: 10.2196/89318 (PMC13563033; doi:10.2196/89318)
Supplement: Supplementary file 1 [file resprot_v15i1e89318_app1.pdf]

PREPS-21-0108

Grille : AAP21-PREPS-grilleExpert-01

Approche de l'investigation / Structuration of the project

1.1 . L'état de l'art du domaine abordé par le projet, les hypothèses et les objectifs principal et secondaires qui en découlent sont décrits ET sont liés de manière cohérente. Vous êtes : :

The description of the current knowledge, the hypotheses and the primary and secondary objectives are adequate; these items are logically linked together. You are : :

plutôt en accord / somewhat agree

1.2 . Les critères d'évaluation principal et secondaires correspondent aux objectifs principal et secondaires ET sont décrits de manière précise [Note : critères d'évaluation = critères de jugement] Vous êtes : :

The primary and secondary endpoints are precisely described AND fit with the primary and secondary objectives [Note: Evaluation criteria = endpoints] You are : :

très en accord / strongly agree

1.3 . Les méthodes de mesure des critères d'évaluation principal et secondaires sont décrites dans le projet. Vous êtes : :

The assessment criteria of the primary and secondary endpoints are well described. You are : :

très en accord / strongly agree

1.4 . Les critères de sélection (inclusion, non inclusion et exclusion) des sujets de la recherche sont décrits ET cohérents avec la population cible du projet. Vous êtes : :

Inclusion, non-inclusion and exclusion criteria are well described and fit the targeted population. You are : :

très en accord / strongly agree

1.5 . Commentaire Approche de l'investigation [commentaire facultatif] :

Structuration of the project comment [optional comment] :

Méthodologie / Methodology

2.1 . Les méthodes de l'investigation sont décrites et adéquates. [Calendriers de l'étude, circuits -patient, données, médicament, biologie, …-, organisation des ressources humaines mobilisées pour la recherche et autres moyens techniques] Vous êtes : :

Study design, conduct and logistics are well described and appropriate: monitoring schedule, procedures and data collection and research governance for patients, data, drugs and biomarkers; human and technical resources organization needed for this research. You are : :

très en accord / strongly agree

2.2 . Le plan expérimental est adapté ET en lien avec la question de recherche posée. Vous êtes : :

The experimental design is appropriate AND linked to the scientific question asked. You are : :

plutôt en accord / somewhat agree

2.3 . Le nombre de sujets à inclure est estimé à partir du critère d'évaluation principal selon une méthode adaptée ET sur la base d'hypothèses de calcul justifiées. Vous êtes : :

The number of subjects to be included is based on the primary endpoint using an appropriate method AND this number is based on justified calculation hypotheses. You are : :

totalement en accord / totally agree

2.4 . Commentaire méthodologie [commentaire facultatif] :

Methodology comment [optional comment] :

Faisabilité / Feasibility

3.1 . La capacité de recrutement des centres participants est présentée, justifiée et adéquate, y compris en lien avec le calendrier du projet. Vous êtes : :

The accrual capacity of participating centers is outlined; it is realistic and fits the trial schedules You are : :

plutôt en désaccord avec cette affirmation/ somewhat disagree with this statement.

3.2 . L'expertise de l'équipe coordinatrice dans le domaine du projet est documentée. Vous êtes : :

The expertise of the coordinating investigator team in the field of the project is well described. You are : :

plutôt en accord / somewhat agree

3.3 . L'équipe coordinatrice et les structures auxquelles elle fait appel ont la capacité de mener à son terme le projet Vous êtes : :

The coordinating investigator and his environment make it likely that the project will succeed. You are : :

plutôt en désaccord avec cette affirmation/ somewhat disagree with this statement.

3.4 . Les moyens annoncés mobilisés et demandés permettent de conduire le projet à son terme [Note : y compris les moyens du contrôle qualité] Vous êtes : :

Required resources (if funded) allow to carry it to successfully complete the proposed project You are : :

plutôt en accord / somewhat agree

3.5 . Commentaire Faisabilité [commentaire facultatif] :

Feasibility comment [optional comment] :

l'expertise de l'équipe pour conduire l'étude n'est pas présentée.

3.6 . Au plan éthique, le projet peut vous poser problème ET/OU sa mise en œuvre pourrait poser problème. [Note : sans préjudice de l'avis qui pourra être rendu par le CPP ou un comité d'éthique] Votre avis :

The approach to ethics approval is not properly described and/or the project is ethically a problem for you. your opinion : :

Oui, cela pose problème / yes, It would raise concerns

3.7 . Commentaire sur le plan éthique [commentaire facultatif] :

Ethics aspects comment [optional comment] :

Appréciation générale / Overall comment

4.1 . Appréciation générale [commentaire obligatoire] :

Overall comment [mandatory comment] :

Section 2.1 L’état actuel des connaissances est pertinent et cohérent. Toutefois plusieurs passages mériteraient d’être davantage soutenus par de la littérature. Par exemple, lorsque l’auteur mentionne que la consommation d’antihypertenseurs a diminué. Quels droits existent mais que la non-demande est fréquente. Que veut-il dire ? Serait-ce possible d’apporter des statistiques, des exemples ? Il faut alors le croire sur parole sans appui. Section 2.2 Comment l’auteur fait-il pour affirmer que la crois-rouge et l’AMM ne font pas de dépistage. Il serait intéressant que l’auteur propose dans la section 2.2. la trajectoire de soins des personnes habitants les zones prioritaires de la CACI afin que nous puissions juger de la véracité de ces propos (absence de service de dépistage, réorientation vers la PASS). Il pourrait également présenter les offres de services et rôle de la croix rouge et de l’AMM afin que nous comprenions qu’aucun dépistage systématique n’est fait. L’hypothèse de la recherche est pertinente. Je suis cependant surpris que l’auteur s’appuie sur des données de 1979 pour soutenir l’importance et l’effet positif du dépistage précoce. Mais encore, qu’en est-il du mécanisme de détection précoce en situation de vulnérabilité ? Mais encore, qu’en est-il de l’efficacité réelle sur la diminution de l’incidence de l’AVC et des maladies cardiovasculaire sur une temporalité longitudinale ? De nombreuses études de synthèse Cochrane ont été réalisées sur le sujet ce qui aurait démontré davantage la rigueur scientifique qui soutient l’hypothèse de recherche du projet. L’hypothèse de la recherche postule que, grâce à une stratégie de dépistage précoce de l’hypertension basée sur « l’aller vers » par des relais communautaires, il sera possible d'augmenter l'efficacité de la prévention primaire auprès des publics précaires (prises en charge précoces, accès aux droits, amélioration du suivi thérapeutique, réduction des renoncements et des interruptions de traitement). Section 2.3 Des références sont nécessaires pou appuyer le recourt au devis quasi-expérimentale de type avant-après. L’auteur n’a pas démontré que la période de 2 ans sera suffisante pour capter l’AVC. Il aurait été pertinent soit en introduction ou en méthode d’amener des données qui présentent le délai de survenu moyen d’un AVC et relié à l’HTA. 2.6 Justifier le recours de la technique d’une mesure unique pour le diagnostic d’hypertension artérielle. Le recours à la mesure unique, en contexte de rencontre médicale, peut induire un biais de diagnostic et de mesure. Est-ce qu’une mesure répétée sur 30 minutes par exemple (en utilisant la moyenne) ne serait-elle pas plus valide (suggestion ici basée sur les guidelines de dépistage et traitement de l’hypertension). Quels sont les éléments qui seront mis en place pour s’assurer de la validité de la mesure diagnostique à une seule mesure ? 3.1. Comment l’objectif de -30% a été établi ? 6.3 Comment les participants potentiels seront recrutés ? Comment donneront-ils leur consentement ? Je comprends les recruteurs approcherons les personnes ? La faisabilité du recrutement pourrait être davantage explicitée. Combien de journées de clinique par mois ? Comment de patients consultent actuellement. On identifie une fréquentation potentielle de 2084 patients potentiels par moi mais nous ne sommes pas convaincus qu’ils viendront consultés. D’autant plus qu’il est connu que les personnes en situation de vulnérabilités ont des habitudes de consultations qui sont différentes. Quelles seront les stratégies pour favoriser la fréquentation de la clinique en lien avec le recrutement ? De plus quel est le taux de refus envisagés. A-t-il été prévu dans les 24 mois. Un recrutement clinique de 50 000 personnes en 24 mois me semble difficilement atteignable. Sur quels travaux antérieurs se basent les auteurs pour justifier ce nombre dans ce délai ? 7.1 et section 9.3.2 Il est décrit dans cette section l’intervention hygiénodiététique en lien avec l’HTA ainsi que les spécificités liées à la maladie. Sur quoi est basé cette formation et enseignement. Quelles sont les lignes directrices utilisées

pour construire cette formation et les enseignements aux patients ? Cette question est également de mise sur les lignes directrices qui guide le diagnostic suite à une mesure unique. 8.1 Est-ce que la prise de médication adéquate sera questionnée aux différentes mesures? 9.9. Est-ce que les décès seront recueillis ? Comment seront-il traités (en lien ou non avec la condition HTA). Comment seront traités les participants qui abandonnent ou qu'on perd de vue ? Comment seront traitées les données manquantes le cas échéant ? 9.10 Il serait intéressant de documenter le nombre d'interventions et référencement à la PASS pour des fins de transférabilité de cette trajectoire de soins au terme du projet (et ainsi prévoir des ressources en conséquence). 10.2 Outre la comparaison selon les quartiers, la couverture sociale, le plan d'analyse de décrit pas si des analyses de sous-groupes (selon certaines caractéristiques) seront conduites. Le traitement des données pour abandon, perdu de vue ou décès n'est pas explicité. 14. Considération éthique en lien avec les personnes en situation de vulnérabilité n'est pas discutée.

Observations sur la grille d'expertise / Comments on the evaluation template

5.1 . Afin d'améliorer la qualité de l'expertise, merci d'indiquer vos commentaires et/ou suggestions d'amélioration sur le fond et/ou la forme de la grille d'expertise [commentaire facultatif] :

In order to improve the quality of the expertise, please indicate your comments and / or suggestions for improvement on the form and/or the substance of the expertise template [optional comment] :
