# Supplementary material for: Community-Based Hypertension Screening to Prevent Stroke in Underserved Urban Neighborhoods in French Guiana: Protocol for a Quasi-Experimental Study
Source: JMIR Res Protoc. 2026 Aug 27;15:e89318. doi: 10.2196/89318 (PMC13563033; doi:10.2196/89318)
Supplement: Supplementary file 2 [file resprot_v15i1e89318_app2.pdf]

PREPS-21-0108

Grille : AAP21-PREPS-grilleExpert-02

Approche de l'investigation / Structuration of the project

1.1 . L'état de l'art du domaine abordé par le projet, les hypothèses et les objectifs principal et secondaires qui en découlent sont décrits ET sont liés de manière cohérente. Vous êtes : :

The description of the current knowledge, the hypotheses and the primary and secondary objectives are adequate; these items are logically linked together. You are : :

totalement en accord / totally agree

1.2 . Les critères d'évaluation principal et secondaires correspondent aux objectifs principal et secondaires ET sont décrits de manière précise [Note : critères d'évaluation = critères de jugement] Vous êtes : :

The primary and secondary endpoints are precisely described AND fit with the primary and secondary objectives [Note: Evaluation criteria = endpoints] You are : :

totalement en accord / totally agree

1.3 . Les méthodes de mesure des critères d'évaluation principal et secondaires sont décrites dans le projet. Vous êtes : :

The assessment criteria of the primary and secondary endpoints are well described. You are : :

totalement en accord / totally agree

1.4 . Les critères de sélection (inclusion, non inclusion et exclusion) des sujets de la recherche sont décrits ET cohérents avec la population cible du projet. Vous êtes : :

Inclusion, non-inclusion and exclusion criteria are well described and fit the targeted population. You are : :

très en accord / strongly agree

1.5 . Commentaire Approche de l'investigation [commentaire facultatif] :

Structuration of the project comment [optional comment] :

Le projet est très convaincant scientifiquement et hautement pertinent de point de vue sociale. Je pense même que les connaissances produites pourront être transférées vers des contextes facilement, malgré le caractère spécifique du lieux d'étude. La question des enjeux de recrutement (usagers et médiateurs) aurait pu être abordée plus directement. Je prends pour acquis que les médiateurs sont compétents à la prise de la TA et qu'ils auront une capacité minimale de navigation vers les ressources cliniques en cas de TA élevée. Je comprends et salue l'approche populationnelle. Les chercheurs auraient pu justifier davantage le choix de ne pas cibler la population avec un facteur de risque clinique, par exemple l'obésité. Je salue positivement l'ampleur du projet, observable par la portée de la structure d'objectifs, qui lie positivement des objets strictement cliniques à des objets organisationnels, voire sociaux. Certains objectifs requerront ou bénéficieraient de compétences en sciences sociales pour bien tirer partie des données collectées. Ces quelques remarques critiques n'entachent en rien mon jugement, très positif sur l'étude.

Méthodologie / Methodology

2.1 . Les méthodes de l'investigation sont décrites et adéquates. [Calendriers de l'étude, circuits -patient, données, médicament, biologie, …-, organisation des ressources humaines mobilisées pour la recherche et autres moyens techniques] Vous êtes : :

Study design, conduct and logistics are well described and appropriate: monitoring schedule, procedures and data collection and research governance for patients, data, drugs and biomarkers; human and technical resources organization needed for this research. You are : :

très en accord / strongly agree

2.2 . Le plan expérimental est adapté ET en lien avec la question de recherche posée. Vous êtes : :

The experimental design is appropriate AND linked to the scientific question asked. You are : :

totalement en accord / totally agree

2.3 . Le nombre de sujets à inclure est estimé à partir du critère d'évaluation principal selon une méthode adaptée ET sur la base d'hypothèses de calcul justifiées. Vous êtes : :

The number of subjects to be included is based on the primary endpoint using an appropriate method AND this number is based on justified calculation hypotheses. You are : :

très en accord / strongly agree

2.4 . Commentaire méthodologie [commentaire facultatif] :

Methodology comment [optional comment] :

voir pemière boîte

Faisabilité / Feasibility

3.1 . La capacité de recrutement des centres participants est présentée, justifiée et adéquate, y compris en lien avec le calendrier du projet. Vous êtes : :

The accrual capacity of participating centers is outlined; it is realistic and fits the trial schedules You are : :

très en accord / strongly agree

3.2 . L'expertise de l'équipe coordinatrice dans le domaine du projet est documentée. Vous êtes : :

The expertise of the coordinating investigator team in the field of the project is well described. You are : :

totalement en accord / totally agree

3.3 . L'équipe coordinatrice et les structures auxquelles elle fait appel ont la capacité de mener à son terme le projet Vous êtes : :

The coordinating investigator and his environment make it likely that the project will succeed. You are : :

totalement en accord / totally agree

3.4 . Les moyens annoncés mobilisés et demandés permettent de conduire le projet à son terme [Note : y compris les moyens du contrôle qualité] Vous êtes : :

Required resources (if funded) allow to carry it to successfully complete the proposed project You are : :

totalement en accord / totally agree

3.5 . Commentaire Faisabilité [commentaire facultatif] :

Feasibility comment [optional comment] :

idem

3.6 . Au plan éthique, le projet peut vous poser problème ET/OU sa mise en œuvre pourrait poser problème. [Note : sans préjudice de l'avis qui pourra être rendu par le CPP ou un comité d'éthique] Votre avis :

The approach to ethics approval is not properly described and/or the project is ethically a problem for you. your opinion : :

non, cela ne pose pas de problème / no, It would raise no concerns.

3.7 . Commentaire sur le plan éthique [commentaire facultatif] :

Ethics aspects comment [optional comment] :

je prends pour acquis que le projet sera soumis à un certificat d'éthique.

Appréciation générale / Overall comment

4.1 . Appréciation générale [commentaire obligatoire] :

Overall comment [mandatory comment] :

Excellent à projet à tous égards, ou presque. Projet fondamentalement de soins primaires, liant intelligemment prévention et intervention. Travail à la fois ambitieux et utile, avec un fort potentiel d'impact clinique.

Observations sur la grille d’expertise / Comments on the evaluation template

5.1 . Afin d'améliorer la qualité de l'expertise, merci d'indiquer vos commentaires et/ou suggestions d'amélioration sur le fond et/ou la forme de la grille d'expertise [commentaire facultatif] :

In order to improve the quality of the expertise, please indicate your comments and / or suggestions for improvement on the form and/or the substance of the expertise template [optional comment] :

Très clair
