# Supplementary material for: Community-Based Hypertension Screening to Prevent Stroke in Underserved Urban Neighborhoods in French Guiana: Protocol for a Quasi-Experimental Study
Source: JMIR Res Protoc. 2026 Aug 27;15:e89318. doi: 10.2196/89318 (PMC13563033; doi:10.2196/89318)
Supplement: Supplementary file 3 [file resprot_v15i1e89318_app3.pdf]

PREPS-21-0108

Grille : AAP21-PREPS-grilleExpert-03

Approche de l'investigation / Structuration of the project

1.1 . L'état de l'art du domaine abordé par le projet, les hypothèses et les objectifs principal et secondaires qui en découlent sont décrits ET sont liés de manière cohérente. Vous êtes : :

The description of the current knowledge, the hypotheses and the primary and secondary objectives are adequate; these items are logically linked together. You are : :

totalement en accord / totally agree

1.2 . Les critères d'évaluation principal et secondaires correspondent aux objectifs principal et secondaires ET sont décrits de manière précise [Note : critères d'évaluation = critères de jugement] Vous êtes : :

The primary and secondary endpoints are precisely described AND fit with the primary and secondary objectives [Note: Evaluation criteria = endpoints] You are : :

totalement en accord / totally agree

1.3 . Les méthodes de mesure des critères d'évaluation principal et secondaires sont décrites dans le projet. Vous êtes : :

The assessment criteria of the primary and secondary endpoints are well described. You are : :

totalement en accord / totally agree

1.4 . Les critères de sélection (inclusion, non inclusion et exclusion) des sujets de la recherche sont décrits ET cohérents avec la population cible du projet. Vous êtes : :

Inclusion, non-inclusion and exclusion criteria are well described and fit the targeted population. You are : :

très en accord / strongly agree

1.5 . Commentaire Approche de l'investigation [commentaire facultatif] :

Structuration of the project comment [optional comment] :

The author presented an excellent introduction and background that positioned the study within relevant international literature on health equity and adverse consequences of not intervening on hypertensive vulnerable populations. The research hypothesis is clearly formulated - the researchers want to verify the impact of a community based intervention. There is one main and eight secondary objectives that are clearly formulated. Furthermore, in section 4.1 of the protocol, the authors clearly illustrate the main measures for each research objective. This study mainly relies on survey data and hospital-administrative data to address the research questions. It is a population based study with very minimal inclusion/exclusion criteria. I understand this in an "incidence study" that is identifying new cases of people that develop severe complications due to hypertension. However, the authors did not mention what they would do for newly identified cases that were not previously managed by the local health system.

Méthodologie / Methodology

2.1 . Les méthodes de l'investigation sont décrites et adéquates. [Calendriers de l'étude, circuits -patient, données, médicament, biologie, …-, organisation des ressources humaines mobilisées pour la recherche et autres moyens techniques] Vous êtes : :

Study design, conduct and logistics are well described and appropriate: monitoring schedule, procedures and data collection and research governance for patients, data, drugs and biomarkers; human and technical resources organization needed for this research. You are : :

totalement en accord / totally agree

2.2 . Le plan expérimental est adapté ET en lien avec la question de recherche posée. Vous êtes : :

The experimental design is appropriate AND linked to the scientific question asked. You are : :

totalement en accord / totally agree

2.3 . Le nombre de sujets à inclure est estimé à partir du critère d'évaluation principal selon une méthode adaptée ET sur la base d'hypothèses de calcul justifiées. Vous êtes : :

The number of subjects to be included is based on the primary endpoint using an appropriate method AND this number is based on justified calculation hypotheses. You are : :

très en accord / strongly agree

2.4 . Commentaire méthodologie [commentaire facultatif] :

Methodology comment [optional comment] :

The authors proposed a quasi-experimental before after and time series experiment that is totally appropriate to address their research questions. The authors present a strong argument for an exhaustive population-based study - 50,000 target population in 2 years seems to be ambitious. It would be great if they can pull this

off with their research team.

Faisabilité / Feasibility

3.1 . La capacité de recrutement des centres participants est présentée, justifiée et adéquate, y compris en lien avec le calendrier du projet. Vous êtes : :

The accrual capacity of participating centers is outlined; it is realistic and fits the trial schedules You are : :

plutôt en accord / somewhat agree

3.2 . L'expertise de l'équipe coordinatrice dans le domaine du projet est documentée. Vous êtes : :

The expertise of the coordinating investigator team in the field of the project is well described. You are : :

totalement en accord / totally agree

3.3 . L'équipe coordinatrice et les structures auxquelles elle fait appel ont la capacité de mener à son terme le projet Vous êtes : :

The coordinating investigator and his environment make it likely that the project will succeed. You are : :

totalement en accord / totally agree

3.4 . Les moyens annoncés mobilisés et demandés permettent de conduire le projet à son terme [Note : y compris les moyens du contrôle qualité] Vous êtes : :

Required resources (if funded) allow to carry it to successfully complete the proposed project You are : :

totalement en accord / totally agree

3.5 . Commentaire Faisabilité [commentaire facultatif] :

Feasibility comment [optional comment] :

The authors present a very detailed recruitment plan where the investigation team shall place information flyers/booklets in the neighborhood of the target population that announces the day and time of free clinical examination of the intervention. However, it is not clear in the protocol where the community outreach workers would effectively carry out the clinical exam of research participants (is it in a school, community health centre? etc..). Second, the authors did not clearly indicate the engagement of local community leaders to mobilize their local communities to participate in the study. Third, it is clear if community outreach workers shall go door to door to identify potential homebound participants or people living with loss of functional autonomy that may not be able to attend the schedule screening meeting.

3.6 . Au plan éthique, le projet peut vous poser problème ET/OU sa mise en œuvre pourrait poser problème. [Note : sans préjudice de l'avis qui pourra être rendu par le CPP ou un comité d'éthique] Votre avis :

The approach to ethics approval is not properly described and/or the project is ethically a problem for you. your opinion : :

non, cela ne pose pas de problème / no, It would raise no concerns.

3.7 . Commentaire sur le plan éthique [commentaire facultatif] :

Ethics aspects comment [optional comment] :

The authors argue that it is a minimal risk research since the only thing the intervention group does is to take the blood pressure of clients and carry out health information sessions. This seems right. However it is not clear how the confidentiality of client information shall be secured in this project.

Appréciation générale / Overall comment

4.1 . Appréciation générale [commentaire obligatoire] :

Overall comment [mandatory comment] :

Overall, I think that this is a very strong grant application. The applicants clearly identified a research-practice gap in the lack of a proactive community-based monitoring program for hypertension in the Guyanese context. They proposed a simple intervention - using trained community outreach workers to roll out the community screening program. They intend to partner with local NGOs - this improves their capacity to achieve the project. The scientific team demonstrated that they clearly mastered international and local literature relevant to their research questions. Apart from minor lack of clarity in the recruitment approach, I find that this proposal is relevant, scientific and has the potential to substantially improve the health of vulnerable local populations.

Observations sur la grille d’expertise / Comments on the evaluation template

5.1 . Afin d'améliorer la qualité de l'expertise, merci d'indiquer vos commentaires et/ou suggestions d'amélioration sur le fond et/ou la forme de la grille d'expertise [commentaire facultatif] :

In order to improve the quality of the expertise, please indicate your comments and / or suggestions for improvement on the form and/or the substance of the expertise template [optional comment] :

The online expertise template is awesome! Actually, this is the first time I am using such a simplified platform. I had not difficulty in navigating the platform.
